# Age at First RSV Hospitalisation and the Risk of Subsequent Bacterial Pneumonia

**DOI:** 10.1111/apa.70481

**Published:** 2026-02-16

**Authors:** Samuel Videholm, Karin Strandell, Maria Björmsjö, Andreas Tornevi, Sven Arne Silfverdal

**Affiliations:** ^1^ Department of Clinical Sciences, Pediatrics Umeå University Umeå Sweden; ^2^ Department of Epidemiology and Global Health, Faculty of Medicine Umeå University Umeå Sweden

**Abbreviations**
CI: confidence interval
ICD‐10: International Classification of Diseases, Tenth Revision
IRR: incidence rate ratio
RSV: respiratory syncytial virus

## 1 Introduction

The global burden of respiratory syncytial virus (RSV) is estimated to be approximately 33 million episodes, 3.6 million hospital admissions and 101 400 deaths in children younger than 5 years [1]. Early‐life RSV infection is associated with subsequent respiratory sequelae, including asthma and pneumonia [2]. In children hospitalised for RSV, the risk of subsequent asthma increases with age at RSV hospitalisation [3]. By contrast, less is known about the association between age at RSV hospitalisation and subsequent pneumonia. Therefore, the aim of this study was to examine the association between age at first RSV hospitalisation and the risk of subsequent bacterial pneumonia in the first 5 years of life.

## 2 Methods

The study included children born in Sweden between 1998 and 2015, with follow‐up until December 2020. The Medical Birth Register was combined with the National Inpatient Register, the Longitudinal Integration Database for Health Insurance and Labour Market Studies, the Cause of Death Register and the Total Population Register. The study was approved by the Regional Ethics Board in Umeå (number 2012–265‐31 M, with amendments 2013–320‐32 M, 2016–353‐32 M and 2017–399‐32 M) and by the Swedish Ethical Review Authority (numbers 2021–06337‐02 and 2023–04444‐02).

Information on RSV and bacterial pneumonia hospitalisations was obtained from the National Inpatient Register. The exposure, age at first RSV hospitalisation, was defined using the following International Classification of Diseases, Tenth Revision (ICD‐10) codes: J12.1, J20.5, J21.0 and B97.4. The outcome, first hospitalisation for bacterial pneumonia, was defined using the following ICD‐10 codes: J13, J14, J15, J16.0, J85.1 and J86.

The risk of bacterial pneumonia was highest in the first months following RSV hospitalisation (Figure S1). Accordingly, we created an exposure variable by combining age at first RSV hospitalisation, a time‐dependent covariate categorised as no previous RSV hospitalisation (no RSV), 0–5 months (age 0–5 MO), 6–11 months (age 6–11 MO), 12–17 months (age 12–17 MO) and 18–23 months (age 18–23 MO), with time since RSV hospitalisation, a time‐dependent covariate categorised as 0–2 months (time RSV 0–2 MO) and ≥ 3 months (time RSV ≥ 3 MO).

We estimated crude and adjusted associations between age at RSV hospitalisation and subsequent bacterial pneumonia using piece‐wise exponential regression models with robust standard errors. Results were presented as crude hazard ratios (HR) with 95% confidence intervals (CI). The crude analysis was controlled for age in years. The adjusted analysis was additionally controlled for potential confounding factors identified using existing knowledge, previous studies [4, 5] and a directed acyclic graph (Figure S2). Specifically, the adjusted analysis was controlled for sex, gestational age (categorised as 22–27, 28–31, 32–36, 37–41 and ≥ 42 weeks), small for gestational age, congenital heart defects (ICD‐10 codes Q20–Q28), oesophageal atresia (ICD‐10 code Q39), Down syndrome (ICD‐10 code Q90), neonatal respiratory conditions in full‐term children (ICD‐10 codes P22–P24), pneumococcal conjugate vaccine (PCV) status at 3 months of age (a time‐dependent variable created by combining information on the date of birth and county of residence, and categorised as no‐PCV, PCV7, PCV10 and PCV13), maternal age at delivery, maternal smoking during pregnancy, parity, maternal education level (categorised as ≤ 9, 10–12, 13–14 and ≥ 15 years), maternal county of residence, seasonality (month of birth) and year of birth. All analyses were performed using Stata version 17 (StataCorp LLC).

## 3 Results

The Medical Birth Register included 1 828 430 children. We excluded 186 684 children with missing data, leaving 1 641 746 children (90% of the original cohort). Children were followed until their first hospitalisation for bacterial pneumonia (*n* = 8327), death (*n* = 4182), emigration (*n* = 23 828) or until 5 years of age. The study included 29 046 RSV hospitalisations and 8 111 046 person‐years of follow‐up time.

Table 1 presents the association between age at first RSV hospitalisation and subsequent bacterial pneumonia. Compared with children who were not hospitalised for RSV, the risk of bacterial pneumonia increased with age at first RSV hospitalisation. For example, children aged 0–5 months at first RSV hospitalisation were around five times (adjusted HR 5.48; 95% CI 3.93–7.64) more likely to be hospitalised for bacterial pneumonia within the first 3 months following RSV hospitalisation and around two times (adjusted HR 1.77; 95% CI 1.53–2.04) more likely thereafter. By contrast, children aged 18–23 months at first RSV hospitalisation were eight times (adjusted HR 8.62; 95% CI 4.23–17.55) more likely to be hospitalised for bacterial pneumonia within the first 3 months following RSV hospitalisation and four times (adjusted HR 4.36; 95% CI 2.79–6.81) more likely thereafter.

**TABLE 1:** Age at first RSV hospitalisation and the risk of subsequent bacterial pneumonia hospitalisation.

	Crude HR (95% CI)	Adjusted HR (95% CI)
No RSV	1 (ref)	1 (ref)
Time RSV 0–2 MO and Age 0–5 MO	6.98 (5.02–9.71)	5.48 (3.93–7.64)
Time RSV 0–2 MO and Age 6–11 MO	10.79 (6.50–17.92)	7.67 (4.61–12.78)
Time RSV 0–2 MO and Age 12–17 MO	9.38 (4.87–18.04)	6.30 (3.25–12.20)
Time RSV 0–2 MO and Age 18–23 MO	13.41 (6.70–26.86)	8.62 (4.23–17.55)
Time RSV ≥ 3 MO and Age 0–5 MO	2.20 (1.91–2.53)	1.77 (1.53–2.04)
Time RSV ≥ 3 MO and Age 6–11 MO	3.17 (2.46–4.08)	2.24 (1.73–2.89)
Time RSV ≥ 3 MO and Age 12–17 MO	6.70 (4.98–9.01)	4.60 (3.40–6.22)
Time RSV ≥ 3 MO and Age 18–23 MO	6.86 (4.51–10.43)	4.36 (2.79–6.81)

*Note:* Analyses included 1 641 746 children, 29 046 RSV hospitalisations (0–5 months, *n* = 20 488; 6–11 months, *n* = 4938; 12–17 months, *n* = 2162; 18–23 months, *n* = 1458), and 8327 bacterial pneumonia hospitalisations.

Abbreviations: CI, confidence interval; HR, hazard ratio; MO, month; RSV, respiratory syncytial virus.

## 4 Discussion

We found that the risk of bacterial pneumonia increased with age at first RSV hospitalisation. A stronger immune response to RSV infection in older children may explain this association. For example, older children (6–24 months of age) with severe RSV infection have a higher expression of neutrophil‐related genes [6]. Importantly, older children are not protected against RSV by current preventive strategies, that is, maternal vaccination and long‐acting monoclonal antibodies administered at birth [2].

To our knowledge, our study is the first large cohort study to examine how age at RSV hospitalisation is associated with subsequent bacterial pneumonia. A limitation is that we were unable to adjust our analyses for some potential confounders, for example, comorbidities diagnosed after the neonatal period, day‐care and breastfeeding. Importantly, the proportion of RSV‐hospitalised children with comorbidities increases with age [7]. These children may also be more susceptible to bacterial pneumonia. Consequently, the observed association between age at RSV hospitalisation and subsequent bacterial pneumonia may, at least in part, reflect an increased underlying susceptibility to respiratory infections.

In conclusion, we found a strong association between age at RSV infection and subsequent bacterial pneumonia. Accordingly, preventing RSV infections during the first 2 years of life may reduce the risk of bacterial pneumonia in the first 5 years of life.

## Author Contributions

Samuel Videholm conceptualised and designed the study, carried out the initial analyses, and drafted the initial manuscript. Karin Strandell participated in design of the study and contributed to the writing of the manuscript. Maria Björmsjö participated in design of the study and contributed to the writing of the manuscript. Andreas Tornevi participated in design of the study and contributed to the writing of the manuscript. Sven Arne Silfverdal participated in design of the study, coordinated and supervised data collection, and contributed to the writing of the manuscript.

## Funding

This study was supported in part by a research grant from the Investigator‐Initiated Studies Program of MSD, Sweden (to Dr. Videholm). The opinions expressed in this paper are those of the authors and do not necessarily represent those of MSD, Sweden. The study was also supported in part by a research grant from Oskarsfonden (to Dr. Videholm and Strandell) and by grants from the Swedish state under the agreement between the Swedish government and the county councils, the ALF‐agreement (to Dr. Videholm).

## Conflicts of Interest

Dr. Videholm received funding from MSD to his institution during the conduct of the study and has served on advisory boards for pneumococcal vaccines and RSV monoclonal antibodies, paid personally but outside the submitted work. Dr. Björmsjö is involved in a vaccine trial for MSD and has received funding from MSD to her institution but outside the submitted work. Dr. Silfverdal has been involved in vaccine trials for MSD and has participated in advisory board meetings on hexavalent, influenza, meningococcal and pneumococcal vaccines, paid to his institution or personally but outside the submitted work. The other authors have no conflicts of interest relevant to this article to disclose.

## Supporting information


**Figure S1:** Nelson‐Aalen cumulative hazard of bacterial pneumonia hospitalisation by age at first RSV hospitalisation, none (blue), 0–5 months (brown), 6–11 months (grey), 12–17 months (green) and 18–23 months (orange).
**Figure S2:** Directed acyclic graph representing the assumed causal structure. E: exposure (age at first RSV hospitalisation), Y: outcome (bacterial pneumonia hospitalisation), S: sociodemographic factors (maternal age, maternal county of residence and maternal education level), P: pregnancy characteristics (maternal smoking and parity), C: child characteristics (sex, gestational age, small for gestational age, congenital heart defects, oesophageal atresia, Down syndrome, neonatal respiratory conditions in full‐term children, pneumococcal conjugate vaccine status, month of birth and year of birth) and U: unmeasured confounders (comorbidities diagnosed after the neonatal period, day‐care, breastfeeding, multi‐infant birth and genetic factors including asthma in a first‐degree relative).

## Data Availability

The data that support the findings of this study are available from the National Board of Health and Welfare, Statistics Sweden. Restrictions apply to the availability of these data, which were used under licence for this study. Data must be obtained directly from the National Board of Health and Welfare in Sweden https://www.socialstyrelsen.se/ and from Statistics Sweden https://www.scb.se/.
