# Supplementary material for: Age at First RSV Hospitalisation and the Risk of Subsequent Bacterial Pneumonia
Source: Acta Paediatr. 2026 Feb 16;115(6):1298–300. doi: 10.1111/apa.70481 (PMC13159759; doi:10.1111/apa.70481)
Supplement: Supplementary file 1 — Figure S1: Nelson‐Aalen cumulative hazard of bacterial pneumonia hospitalisation by age at first RSV hospitalisation, none (blue), 0–5 months (brown), 6–11 months (grey), 12–17 months (green) and 18–23 months (orange). Figure S2: Directed acyclic graph representing the assumed causal structure. E: exposure (age at first RSV hospitalisation), Y: outcome (bacterial pneumonia hospitalisation), S: sociodemographic factors (maternal age, maternal county of residence and maternal education level), P: pregnancy characteristics (maternal smoking and parity), C: child characteristics (sex, gestational age, small for gestational age, congenital heart defects, oesophageal atresia, Down syndrome, neonatal respiratory conditions in full‐term children, pneumococcal conjugate vaccine status, month of birth and year of birth) and U: unmeasured confounders (comorbidities diagnosed after the neonatal period, day‐care, breastfeeding, multi‐infant birth and genetic factors including asthma in a first‐degree relative). [file APA-115-1298-s001.docx]

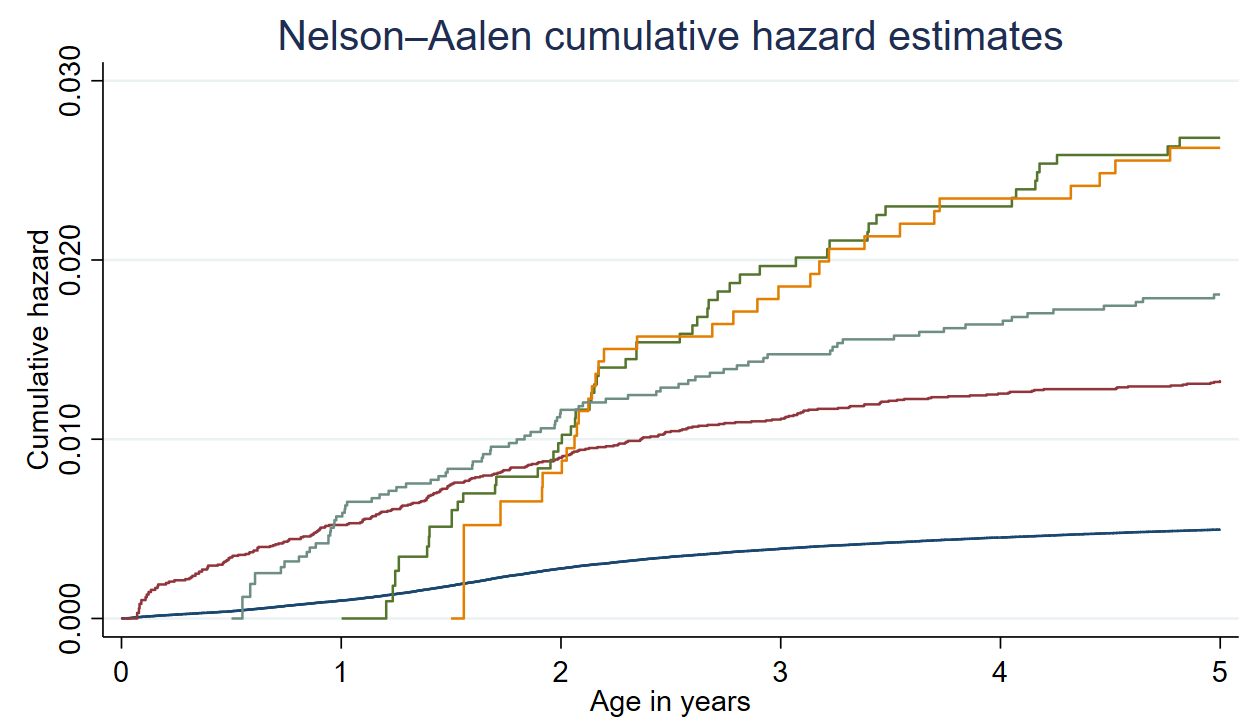
Figure S1: Nelson-Aalen cumulative hazard of bacterial pneumonia hospitalisation by age at first RSV hospitalisation, none (blue), 0–5 months (brown), 6–11 months (grey), 12–17 months (green), and 18–23 months (orange).


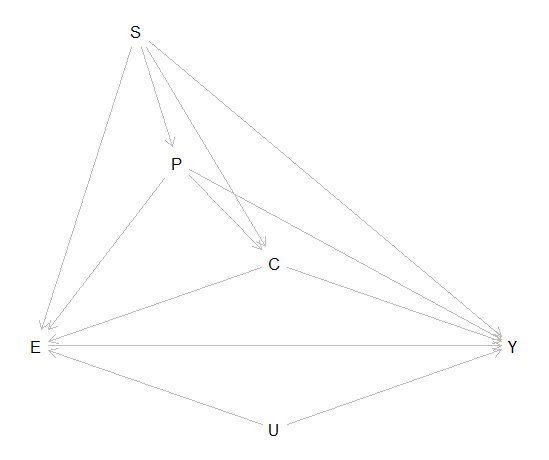


Figure S2: Directed acyclic graph representing the assumed causal structure. E: exposure (age at first RSV hospitalisation), Y: outcome (bacterial pneumonia hospitalisation), S: sociodemographic factors (maternal age, maternal county of residence, and maternal education level), P: pregnancy characteristics (maternal smoking and parity), C: child characteristics (sex, gestational age, small for gestational age, congenital heart defects, oesophageal atresia, Down syndrome, neonatal respiratory conditions in full-term children, pneumococcal conjugate vaccine status, month of birth and year of birth), and U: unmeasured confounders (comorbidities diagnosed after the neonatal period, day-care, breastfeeding, multi-infant birth, and genetic factors including asthma in a first-degree relative).
